# A New Procedure with the Paraffine Method

**Published:** 1888-11

**Authors:** William C. Krauss

**Affiliations:** Attica, N. Y., P. T. University of Berlin; 5 Rue Rollin, Paris


### *A NEW PROCEDURE WITH THE PARAFFINE METHOD.*

By Dr. WILLIAM C. KRAUSS,

Attica, N. Y., P. T. University of Berlin.

(*From Fortschritte der Medicin*, 1888, *No* 16.)

Especially translated for the BUFFALO MEDICAL AND SURGICAL JOURNAL by the Author.

In the early part of the year, I received information of a method in use in the neurological laboratory of Prof. H. Virchow, for preserving the external contour of brains, by means of paraffine infiltration.*

I immediately made use of this method in the laboratory of Prof. Mendel (Berlin), and selected for a trial-specimen the caudate portion of the pons and medulla, possessing a smooth-cut surface. This speci-

NOTE.—The paraffine-infiltration method was first described by Fredericq, afterwards by Schwalbe, in *Anatomischer Anzeiger*, 1886.

men, which had been hardened in seventy-five per cent. alcohol for several weeks, was replaced in ninety-five per cent. alcohol for ten days, to which had been added a small amount of chlorate of calcium. It was then placed in ol. terebinth. pur. for eight days, taken out, and dried by means of filter paper. It was next placed in a molten paraffine mass and subjected to a temperature of 50° Celsius, in a brood oven. At the end of eight days the preparation was removed from the paraffine mass, placed on thick filter paper and replaced in the brood oven for two days with a temperature of 50° Celsius. After allowing the preparation to cool, the smooth-cut surface presented an interesting and instructive appearance. The crura cerebelli ad cerebrum, the fasiculus longitudinalis posterior, and the lemniscus, white nervous matter, were distinctly differentiated from the surrounding gray tissues, while the substantia ferruginea assumed a dark brown color. In other words, with the exception of the pyramids, the white matter of the pons was distinctly and accurately differentiated from the gray, thus showing clearly and perfectly the component structures of the pons. This interesting but merely accidental phenomena led me to make further experiments with this method, paying particular attention to smooth-cut surfaces.

From several brains which had been hardening in seventy-five to ninety per cent. alcohol, I made transverse sections one-quarter, one-half, and one cm. thick through the crura cerebri, pons, and medulla, and placed them for ten days in absolute alcohol, some with and some without the addition of calcium chlorate. After allowing them to dry on filter paper, they were placed in ol. terebinth. pur. for three to five days, until translucent. Instead of using turpentine, benzine may be used, also petroleum.

For further treatment, I found a few modifications necessary. It is well to cover the bottom of the jar containing the paraffine with filter paper, and to place the surface of the section, which one wishes to prepare, downwards. Before placing the sections in the paraffine mass, they must first be well cleared of the turpentine, benzine, or petroleum. I used paraffine with a melting point of 45° C. The temperature of the brood oven may vary between 50° and 60° C. A temperature lighter than this decolorizes the preparation and renders the differentiation between the white and gray matter somewhat indistinct. Should the temperature of the brood oven fall below 45° C., converting the paraffine into a solid mass, the sections are by no means ruined. By regulating the temperature of the brood oven, the process may be continued as before.

After the preparations have been in the brood oven forty-eight to seventy-two hours, they should be removed by means of a spatula and placed on thick filter paper. It is *now* absolutely necessary to place the surface, which one wishes to differentiate, downwards. After subjecting them thirty-six to forty-eight hours to a temperature of 50° to 60° C., in order to remove the superfluous paraffine, and allowing them to cool, the preparations are ready. The surface which has been turned downwards is the one which presents the differentiation. The last stage of the process may be shortened somewhat by subjecting the preparations to a temperature of 75° to 80° C. The superfluous paraffine is quickly removed at this temperature, and after six to eight hours the preparations may be removed and allowed to cool.

With but few exceptions, I obtained good results with the above procedure. Should the differentiation be indistinct, or absent altogether, the preparations should be replaced in the paraffine mass for twenty-four hours and the process completed as before. I have found sections which have withstood two trials to succeed on the third.

The preparations are successful when the white matter is distinctly differentiated from the gray, and this is especially true in those places where, anatomically, the white and gray matter are sharply differentiated.

The gray matter assumes a light-brown color, and in particular places where pigment cells abound, as substantia ferruginea and substantia nigra, a decided darker hue is obtained. The white matter assumes a white, chalky appearance.

Transverse and longitudinal sections through the crura cerebri, pons, and medulla are best adapted for this method. Sections through the hemispheres, especially through the insula, also prove very satisfactory. Transverse sections through the corpora olivarum and further caudate do not prove as satisfactory.

The advantages of these preparations are evidently their adaptation to class demonstration, and as guides in the microscopic and macroscopic examination of the brain. The small amount of shrinkage which takes place does not interfere with the precise anatomical structure of the various parts.

5 Rue Rollin, Paris.

